# Electromagnetic Waves Can Help Improve the Rate of Increase of Milk Feeds Per Day in Premature Infants With Necrotizing Enterocolitis: A Pilot Trial

**DOI:** 10.3389/fped.2022.775428

**Published:** 2022-03-10

**Authors:** Xuexiu Liu, Xianhong Zhang, Luquan Li, Jianhui Wang, Liping Wu

**Affiliations:** ^1^Department of Neonatal Diagnosis and Treatment Center, Children's Hospital of Chongqing Medical University, National Clinical Research Center for Child Health and Disorders, Ministry of Education Key Laboratory of Child Development and Disorders, Chongqing, China; ^2^China International Science and Technology Cooperation Base of Child Development and Critical Disorders, Children's Hospital of Chongqing Medical University, Chongqing, China; ^3^Chongqing Key Laboratory of Pediatrics, National Clinical Research Center for Child Health and Disorders, Ministry of Education Key Laboratory of Child Development and Disorders, Chongqing, China; ^4^Department of Nursing, Children's Hospital of Chongqing Medical University, National Clinical Research Center for Child Health and Disorders, Ministry of Education Key Laboratory of Child Development and Disorders, Chongqing, China

**Keywords:** the rate of increase of milk feeds per day, electromagnetic wave, premature infants, necrotizing enterocolitis, prospective trial

## Abstract

**Objective:**

To evaluate the effects of electromagnetic waves generated by a commercial medical electromagnetic instrument (trade name, TDP, the Chinese phonetic abbreviation of “Te-ding Dian-ci-bo Pu”) as an adjuvant to improve the rate of increase of milk feeds per day by premature infants with necrotizing enterocolitis (NEC).

**Methods:**

This study was a prospective randomized clinical trial. A total of 103 premature infants were diagnosed with NEC II, but there was no need for surgery. The infants were randomly divided into the TDP intervention group and the control group by a randomized method using SPSS 24.0. The patients in the TDP intervention group were treated with TDP irradiation and routine interventions; those in the control group were treated with routine interventions. The rate of increase of milk feeds per day, the time to achieve total gastrointestinal nutrition, the velocity of weight gain, and the complication incidence rate were recorded and compared.

**Results:**

The rate of increase of milk feeds per day in the TDP intervention group was significantly greater than that in the control group [14.51 (11.58~22.11) ml/kg/d vs. 10.15 (6.15~15.87) ml/kg/d, *P* = 0.002]. Compared to the control group, the time to achieve total gastrointestinal nutrition (21.45 ± 1.87 d vs. 36.43 ± 2.585 d, *P* = 0.000) and the velocity of weight gain (19.65 ± 15.27% vs. 13.68 ± 7.15%, *P* = 0.013) in the TDP intervention group were substantially better than those in the control group. The complication incidence rate was not significantly different between the two groups (*P* > 0.05).

**Conclusion:**

Treatment with TDP-generated electromagnetic waves improved the volume of milk consumed per day in premature infants with NEC II and were conducive to improving their clinical outcomes.

## Introduction

Necrotizing enterocolitis (NEC) is a life-threatening intestinal inflammatory disease of new-borns. Pathophysiologically, impeded blood circulation of the intestinal mucosa may play a pivotal role, resulting in intestinal ischaemia or necrosis. Patients with NEC often present with abdominal distention, vomiting, bloody stool, and typical intestinal wall pneumatosis on abdominal radiography (1). NEC occurs in 5–12% of new-borns and is common in preterm and low-birth-weight babies, with high mortality (2, 3). A multicentre study in China showed that the mortality of NEC was ~41.7% among low-birth-weight babies and 50.2% among very-low-birth-weight babies (4). Despite years of effort, the mortality associated with NEC remains high, up to 30%, and is even higher among those treated with surgery (5); if the patients survive, many may face other troublesome conditions, such as peritonitis, intestinal obstruction, and physical and mental delays (6, 7).

Regarding treatment for NEC, mainstream clinical strategies are still supportive care for symptoms, including fasting, gastrointestinal decompression, fluid infusion, and anti-infection (8). There is no way to improve the patient's intestinal circulation. Long-term fasting and gastrointestinal decompression can lead to slow milk consumption and long-term enteral nutrition. It seriously affects the nutritional status of children. NEC is associated with a long hospitalization time and high mortality (9–12), and it has a high incidence rate. Therefore, it is of great significance to introduce more aggressive therapies to increase the volume of milk consumed per day in patients. Studies have shown that vasoactive drugs such as low-dose dopamine can improve intestinal blood circulation, reduce the level of inflammatory factors in children with NEC (13), improve the treatment effect and improve the prognosis.

Electromagnetic waves generated by a commercial medical electromagnetic device (trade name, TDP, the Chinese phonetic abbreviation of “Te-ding Dian-ci-bo Pu”) in China have been reported to serve as an adjuvant physiotherapy for the rehabilitation of a variety of diseases (14). In this study, we used TDP-generated electromagnetic waves as supplementary physiotherapy to irradiate the bellies of children with NEC II and compare the primary clinical outcomes of the TDP irradiation and control groups to determine the value of this treatment for NEC. To the best of our knowledge, few previous studies have focused on this topic.

## Methods

### Participants and Grouping

This study was a single-center, prospective clinical trial. It is registered in the Chinese clinical trial registry, registration number: ChiCTR2000041336. Following the approval of the Ethics Committee of the Children's Hospital of Chongqing Medical University (CHCMU) (No. 2019–289), 110 premature infants who were diagnosed with NEC II and did not need surgery in the neonatal unit of CHCMU were enrolled from December 2020 to August 2021. NEC II was diagnosed based on the Bell classification (9) method according to the International Classification of Disease (11th version). The inclusion criteria were as follows: (1) patients who met the diagnostic criteria for neonatal NEC based on the Bell stage in practical neonatology (1) and were diagnosed as NEC stage II based on laboratory examination and abdominal X-ray plain film examination; (2) patients of gestational age 28–36^+6^ weeks; (3) patients without congenital intestinal malformations, such as congenital intestinal atresia, Hirschsprung's disease, intestinal malrotation and primary intestinal perforation; and (4) patients without blood coagulation disorders. The exclusion criteria were as follows: (1) patients with other infectious diseases; (2) patients with incomplete clinical data; and (3) the patient withdrew from the study from NECII to NECIII, after operation or death.

The qualified patients were randomly grouped using SPSS 24.0 into the TDP intervention group and the control group. The control group was treated with conventional fasting, gastrointestinal decompression, rehydration, and anti-infection, while the TDP intervention group was treated with the abovementioned regimen and TDP. Patient responses to treatment, including the rate of increase of milk feeds per day, time to achieve total gastrointestinal nutrition, velocity of weight gain, and incidence of complications, were recorded.

### TDP Treatment Protocol

**Figure 1:**
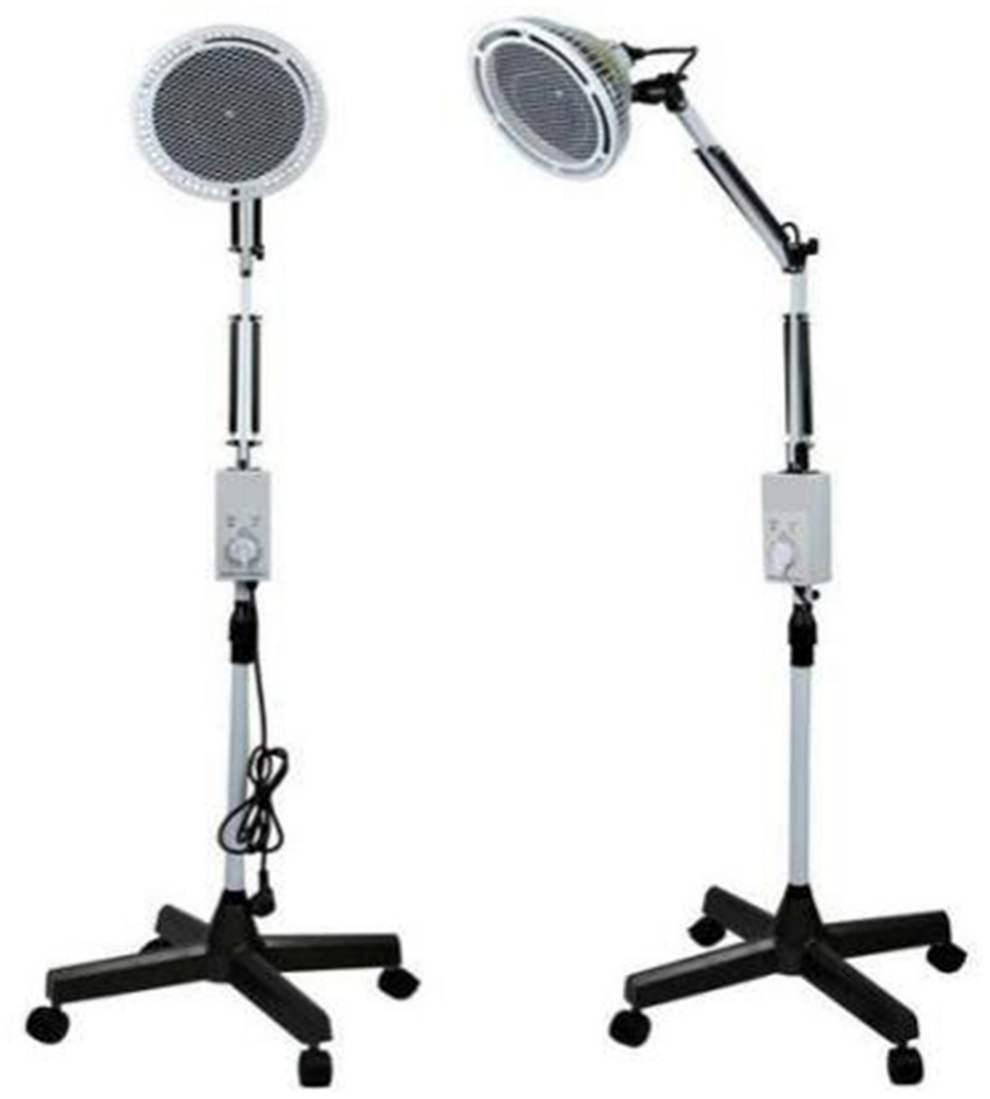
This is a TDP picture.

Electromagnetic waves were generated by three TDP devices (Guoren, Chongqing Guoren Medical Equipment Co., Ltd., Chongqing) as detailed in Figure 1. TDP treatment was initiated immediately after a confirmed diagnosis of NEC II and was performed twice a day with an interval of 5 h by a trained charge nurse after the apparatus was preheated for 5–10 min. The nurse stayed with the child, puting TDP over the patient's abdomen, the radiation part must be completely exposed, otherwise the curative effect will be affected;adjusting the height of the apparatus head to keep a distance of 20–30 cm from the body and the skin surface temperature is maintained at 40 ± 2 degrees to avoid scalding and to ensure that the child's abdomen was exposed under irradiation for 30 min while monitoring vital signs, skin temperature and color, and other problems.

### Sample Size

Based on a previous study (15), we estimated the primary outcomes of the rate of increase of milk feeds per day in the observation group and the control group, and the values were 2.69 (1.92~4.17) and 1.57 (0.81~3.0) ml/d, respectively. Using a bilateral α level of 0.05, we determined that a sample size of at least 86 patients was required for the statistical tests. Because the treatment of NEC is affected not only by TDP irradiation but also by a variety of complex factors and changes in clinical practice, we assumed that a 20% probability was clinically acceptable.

### Statistical Analysis

SPSS 24.0 software (Chicago, IL, USA) was used for data processing and statistical analysis. Quantitative data conforming to a normal distribution are expressed as ([[Inline Image]]± s), and the *T*-test and Mann–Whitney *U*-test were separately used to investigate the intergroup differences. Count data are expressed as ratios or percentages (%), and the chi-square test was applied. *P* < 0.05 indicates a significant difference.

## Results

### Demographic Data

**Figure 2:**
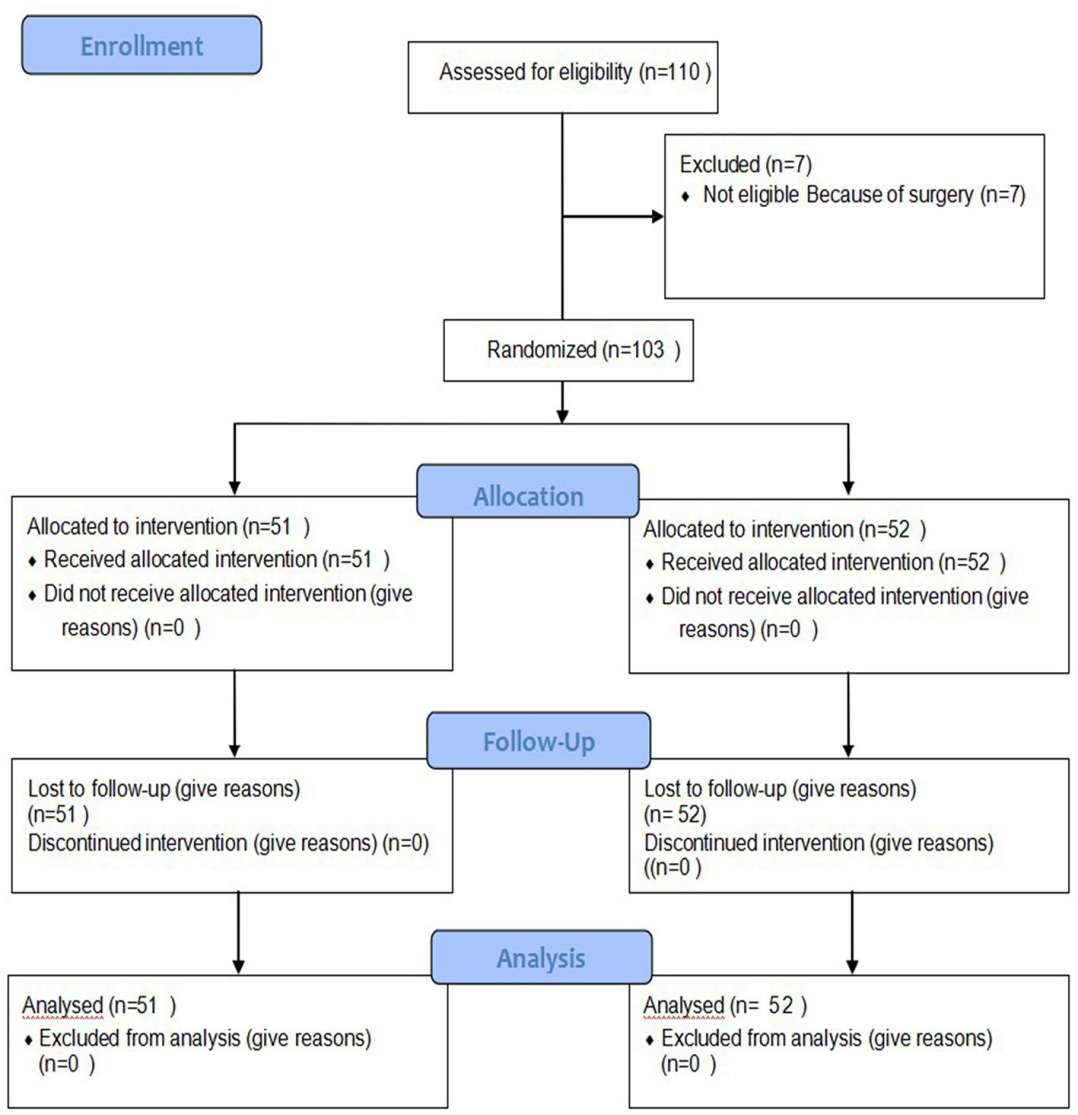
CONSORT Diagram.

A total of 110 premature infant patients were confirmed to have NEC II and had no need for surgery. Among them, seven dropped out of the study for a later surgery, and 103 patients were enrolled in the study. The infants were divided into the TDP intervention group or the control group by a randomized method using SPSS 24.0 as detailed in Figure 2.

At admission, there were no significant differences between the two groups in terms of the baseline data, including sex, birth weight, gestational age, age (days), and APGAR score as detailed in Table 1 (*P* > 0.05 for all variables).

**Table 1:** Comparison of the baseline data between the two groups.

**Variable**	**TDP (*n* = 51)**	**Control (*n* = 52)**	**Statistics**	**95% CI**	***P*-value**
Female **[***n* (%)**]**	21 (41.2)	18 (34.6)	0.374	1.167 (0.711–1.915)	0.541
Amniotic fluid pollution **[***n* (%)**]**	4 (7.8)	10 (19.2)	2.981	0.4 (0.134–1.193)	0.084
Cesarean section **[***n* (%)**]**	34 (66.7)	37 (71.1)	0.417	0.919 (0.711–1.188)	0.518
PROM **[***n* (%)**]**	12 (23.5)	15 (28.8)	0.453	0.8 (0.417–1.536)	0.501
Gestational age (week)	33.92 ± 2.53	33.12 ± 2.65	1.564	(0.215–1.823)	0.121
Birthweight (g)	1,950 (1,546~2,361)	1,998 (1,500~2,270)	1.633	(1,548–2,259)	0.843
Age (day)	7.00 (1.00~17.00)	7.00 (0.084~19.00)	0.255	(5.114–6.503)	0.799
APGAR score at 1 min ≤ 7 **[***n* (%)**]**	9 (17.6)	15 (28.8)	1.962	(0.049–0.284)	0.161
APGAR score at 1 min ≤ 7 **[***n* (%)**]**	2 (3.9)	7 (13.46)	3.047	(0.033–0.281)	0.081
APGAR score at 10 min ≤ 7 **[***n* (%)**]**	0	3 (5.7)	3.091	(0.014–0.181)	0.079
Neonatal sepsis **[***n* (%)**]**	5 (9.8)	6 (11.5)	0.102	(0.391–3.684)	0.750

### Efficacy Indices

Compared to the control group, the TDP intervention group exhibited a significant the rate of increase of milk feeds per day [14.51 (11.58~22.11) vs. 10.15 (6.15~15.87) ml/kg/d, *P* = 0.002] and the velocity of weight gain (19.65 ± 15.27 vs. 13.68 ± 7.15%, *P* = 0.013) and a significant decrease in the time to achieve total gastrointestinal nutrition (21.45 ± 1.87 vs. 36.43 ± 2.585 d, *P* = 0) as summarized in Table 2.

**Table 2:** Comparison between the two groups after treatment between the two groups.

**Indicator**	**TDP (*n* = 51)**	**Control (*n* = 52)**	**Statistics**	**95% CI**	***P*-value**
Increased volume of milk per day (ml/d)	2.69 (1.92~4.17)	1.57 (0.81~3.0)	3.567	(1.365–3.577)	0
The time of achieving total gastrointestinal nutrition (d)	21.45 ± 1.87	36.43 ± 2.585	4.695	(8.651–21.31)	0
The velocity of weight gain [*n* (%)]	19.65 ± 15.27	13.68 ± 7.15	2.556	(1.344–10.672)	0.013

### Safety Variables

Before discharge, no patient died or was burned by TDP irradiation in either group. There were no significant differences in the incidences of neonatal sepsis, neonatal pneumonia, gastrointestinal bleeding, intracranial hemorrhage, gastric wall necrosis or perforation between the two groups (*P* > 0.05 for all variables) as shown in Table 3.

**Table 3:** Comparison of complications and coexisting diseases between the two groups.

**Indicator**	**TDP (*n* = 51)**	**Control (*n* = 52)**	**Statistics**	**95% CI**	***P*-value**
Neonatal sepsis **[***n* (%)**]**	21 (41.2)	28 (53.8)	1.925	0.75 (0.497–1.132)	0.165
Neonatal pneumonia **[***n* (%)**]**	35 (68.6)	42 (80.7)	2.596	0.833 (0.666–1.043)	0.107
Gastrointestinal bleeding **[***n* (%)**]**	4 (7.8)	6 (11.5)	0.443	0.667 (0.2–2.223)	0.505
Intracranial hemorrhage **[***n* (%)**]**	8 (15.7)	7 (13.4)	0.078	1.143 (0.448–2.917)	0.78
Necrosis and perforation of gastric wall [*n* (%)]	1 (2)	0	0	0.98 (0.943–1.019)	1

## Discussion

Electromagnetic waves generated by TDP belong to the near-infrared and middle-infrared spectrum bands, featuring mixed wavelengths of 0.65–50 μm and energy intensities of 25–35 mw/cm^2^ (13). Thus, TDP for medical rehabilitation probably works similarly to other near-infrared and middle-infrared radiation technologies. The actions of near-infrared and middle-infrared radiation on organisms have been well-studied previously (16–19). These forms of radiation penetrate skin and tissues, exerting therapeutic actions, it can promote tissue blood circulation, healing, re-epithelialization, and antibacterial effect (20–23). And can cause the effective expansion of the rat mesenteric microcirculations, resulting in a significant increase in vascular blood flow and promote the recovery of abdominal blood vessels and tissues (24, 25).

NEC is a serious intestinal disease in neonates. It affects the blood supply of the intestinal mucosa, resulting in intestinal wall ischemia and necrosis, seriously affects gastrointestinal function, and affects the prognosis of patients, and the mortality associated with NEC is very high. At present, routine treatment relies on fasting, gastrointestinal decompression, and parenteral nutrition, among other methods. Long-term fasting and gastrointestinal decompression seriously affect the nutritional status of patients.

The rate of increase of milk feeds per day is an independent risk factor for the prognosis of NEC (26, 27). In addition, premature infants are given parenteral nutrition *via* umbilical vein catheterization and peripherally inserted central catheterization. Long-term deep vein catheterization can lead to an increased incidence of infectious diseases. Long-term fasting and gastrointestinal decompression can cause gastrointestinal mucosal atrophy, digestive dysfunction, and feeding intolerance. Therefore, it is very important to feed as early as possible, fast carefully, speed up the rate of increase of milk feeds per day, and reach total gastrointestinal nutrition as soon as possible (28).

Many studies have confirmed that electromagnetic waves can improve the treatment of many diseases (17, 18). When TDP is used to irradiate the abdomen, electromagnetic radiation acts on the intestinal wall to generate heat and accelerate local blood circulation (29), thus increasing the blood supply of the intestinal wall, alleviating ischemia, promoting vasodilation, accelerating blood flow, relaxing spasmodic intestinal vascular smooth muscle, improving capillary permeability and tissue nutrition, promoting metabolism, phagocytosis of leukocytes, absorption and dissipation of inflammatory exudates and departure of inflammatory cells, reducing immune damage and promoting repair of damaged tissues (30). In addition, intestinal peristalsis is slow, and defecation is weak after abdominal distension in patients with NEC. TDP irradiation can improve the muscle state, relax smooth muscle, relieve muscle tension and abdominal distension, promote the recovery of gastrointestinal peristalsis function (17), shorten gastrointestinal emptying time, promote the fecal excretion of new-borns, and greatly reduce the retention time of food residues in new-borns. Compared to the control group, infants in the observation group who were treated with TDP had a significantly the rate of increase of milk feeds per day, gained significantly more body weight, and exhibited a decreased time to achieve total gastrointestinal nutrition. Considering these findings, we believe that TDP irradiation accelerated abdominal blood circulation and promoted the recovery of gastrointestinal function in children with NEC.

There was no significant difference in the incidence of neonatal sepsis, gastrointestinal bleeding or other complications between the two groups, indicating that conventional symptomatic treatment combined with TDP adjuvant treatment will not increase the incidence of adverse reactions in children with NEC.

### Limitations

This study has at least four limitations. First, for safety considerations, only patients with stage II NEC were enrolled in the study, and the therapeutic responses of patients at other stages remain unknown. Second, the study did not compare the effects of different treatment regimens. Third, due to the wide frequency of bands for TDP-generated electromagnetic waves, the frequency bands that play a key role in the TDP effect need to be studied. Fourth, we did not use ultrasound to evaluate the patient's intestinal blood flow. In the next step, we will further use ultrasound to verify it.

## Conclusions

In summary, more aggressive strategies other than supportive treatment are required to improve the clinical outcomes of patients with NEC. The findings of this pilot study showed that TDP irradiation of the abdomen of patients with NEC II is safe and effective. Patients in the TDP intervention group consumed more milk and gained weight more quickly than those in the control group. The therapeutic mechanisms of electromagnetic waves are still unclear. Further experimental studies are needed.

## Data Availability Statement

The original contributions presented in the study are included in the article/supplementary material, further inquiries can be directed to the corresponding author/s.

## Ethics Statement

The studies involving human participants were reviewed and approved by the Ethics Committee at the Children's Hospital of Chongqing Medical University. Written informed consent to participate in this study was provided by the participants' legal guardian/next of kin.

## Author Contributions

XL: contributed to the acquisition, analysis and interpretation of the data and to the drafting and final approval of the manuscript. LW and XZ: provided technical support and conceptual advice. LL and JW: designed the study. All authors read and approved the final manuscript.

## Funding

This work was supported by the Chongqing Science and Technology Commission (cstc2018jscx-msybX0027) of China.

## Conflict of Interest

The authors declare that the research was conducted in the absence of any commercial or financial relationships that could be construed as a potential conflict of interest.

## Publisher's Note

All claims expressed in this article are solely those of the authors and do not necessarily represent those of their affiliated organizations, or those of the publisher, the editors and the reviewers. Any product that may be evaluated in this article, or claim that may be made by its manufacturer, is not guaranteed or endorsed by the publisher.
